# Relationship between online cognition and personality traits: A questionnaire based study of medical college students

**DOI:** 10.1192/j.eurpsy.2021.1538

**Published:** 2021-08-13

**Authors:** N. Ohri, A. Gill, G. Vankar, P. Tyagi, S. Reddy

**Affiliations:** 1 Psychiatry, New Life Hospital, Varanasi, India; 2 Psychiatry, Parul Institute of Medical Scieces, Vadodara, India; 3 Psychiatry, Sri Aurobindo Medical College and PGI, Indore, India

**Keywords:** online cognition scale, process addiction, Internet addiction, personality traits

## Abstract

**Introduction:**

Current classification systems are not sure where to place the internet use disorder. Is it an addiction, an impulse control disorder, a consequence of another psychiatric morbidity or a consequence of personality trait/personality disorder?

**Objectives:**

We intended to study which personality traits associated with online cognition may contribute towards Problematic internet use(PIU). We also analysed the relationship between number of hours of use/week of internet and PIU along with its relation with two ‘screening’ questions.

**Methods:**

Online cognition scale and Abbreviated Eysenck Personality questionnaires were our measurements of choice in addition to demographic measures and some questions pertaining to online behaviour patterns.

**Results:**

Total 163 responses were analysed. The demographic pools consisted mostly of young adults who had, on average, used the internet for 5.2 years at present rate of 21.81hours/week. We observed significantly higher mean OCS scores in men, in people who thought that the internet interfered with their lives and in those who felt the need to ‘cut-down’. A moderate positive and significant correlation was observed between hpurs/week of internet use and OCS scores. Also, significant positive correlation was observed between Neuroticism and OCS, impulsivity, and loneliness/depression scores. Significant negative correlations were observed between the Lie trait and impulse control. Neuroticism and Lie together contributed to 21.8% of variance in OCS scores.

**Conclusions:**

Neuroticism and Lie traits (representing need for social acceptance) were found to the causing significamn varience in the OCS scores of the subjects. High number of hours/week use of internet was related to the feeling of ‘need to cut down use’.

